# A LONGITUDINAL INCREASE IN SLEEP HEALTH PROBLEMS IN MIDDLE ADULTHOOD PREDICTS EARLY MORTALITY

**DOI:** 10.1093/geroni/igac059.1178

**Published:** 2022-12-20

**Authors:** Soomi Lee, Christina Mu, Meredith Wallace, Ross Andel, David Almeida, Orfeu Buxton, Sanjay Patel

**Affiliations:** University of South Florida, Tampa, Florida, United States; University of South Florida, Tampa, Florida, United States; University of Pittsburgh, Pittsburg, Pennsylvania, United States; University of South Florida, Tampa, Florida, United States; The Pennsylvania State University, University Park, Pennsylvania, United States; The Pennsylvania State University, University Park, Pennsylvania, United States; University of Pittsburgh, Pittsburgh, Pennsylvania, United States

## Abstract

Negative consequences of sleep health problems are common in middle-age but poorly understood. This study investigated multidimensional sleep health in middle adulthood and mortality risk. Participants from the Midlife in the United States Study reported sleep characteristics in 2004-2006 (T1; n=9,640, Mage=52.72) and again in 2013-2016 (T2; n=4,334). Deaths since each survey were logged. Multidimensional sleep health composite captured Regularity, Satisfaction, Alertness, Efficiency, and Duration. Cox regression adjusted for sociodemographics and known risk factors (BMI, smoking, depression/anxiety, diabetes, and hypertension) indicated that each unit higher sleep health problems at T1 was associated with 245% and 324% increase in hazard rates for all-cause (Hazard Ratio; HR=3.45, p<.001) and heart disease (HR=4.24, p<.001) mortality, respectively. Those with an increase in sleep health problems at T2 compared to T1 had a 182% increase in all-cause mortality risk (HR=2.82, p<.05), but not heart disease mortality risk. Improving sleep health may reduce early mortality risk.

